# Changes in the nutritional content of children's lunches after the Food Dudes healthy eating programme

**DOI:** 10.1017/jns.2021.31

**Published:** 2021-05-31

**Authors:** Mariel Marcano-Olivier, Jake Sallaway-Costello, Lorna McWilliams, Pauline J. Horne, Simon Viktor, Mihela Erjavec

**Affiliations:** 1 School of Social Sciences, Birmingham City University, 4 Cardigan Street, Birmingham B4 7BD, UK; 2 Department of Therapies and Public Health, University of Nottingham, University Park Campus, Nottingham NG7 2RD, UK; 3 Centre for Health Psychology, University of Manchester, 46 Grafton Street, Manchester M13 9NT, UK; 4 School of Psychology, Bangor University, Brigantia, Penrallt Road, Bangor (Gwynedd) LL57 2AS, UK

**Keywords:** Children: Consumption, Food Dudes, Fruit and vegetables, Healthy eating, Macronutrients, Micronutrients, School lunch

## Abstract

Previous research into the effectiveness of healthy eating programmes has shown increases in healthful eating behaviour in primary schools; however, data collection methods have not been sufficiently sensitive to detect micronutrient changes. The present study extends the literature by measuring individual children's intake of macro- and micronutrients at lunchtime, before and after a programme targeting children's consumption of fruit and vegetables, to identify evidence-based health benefits of programme participation. Baseline data were collected over 4 d at lunchtime in two primary schools. The Food Dudes programme was then implemented in the intervention school. Follow-up data were collected over 4 d in each school 2 months after baseline. We employed a validated and sensitive photographic method to estimate individual children's (*N* 112) consumption of fruit, vegetables, and their intake of calories, macro- and selected micronutrients. Significant changes were observed in the intervention school but not in the control school: Children's consumption of fruit, vegetables, vitamin C and E intake increased, while their total energy consumption, fat, saturated fat, and sodium intake decreased. The present results show that the Food Dudes programme produced a positive nutritional change, with implications for its application as a healthy eating and obesity prevention intervention. These optimistic conclusions should be tested by further research to establish the longevity of the positive effects presented here.

## Introduction

Children in most developed countries, including the UK, over consume foods high in fats, sugar and salt and do not eat enough fruit and vegetables, which increases their risk of ill health^(1)^. Data gathered by the National Child Measurement Programme^(2)^ suggest that approximately one-third of children in England are either overweight or obese, with the prevalence of obesity more than doubling between reception year (ages 4–5) and year 6 (ages 10–11) pupils (9⋅1 and 19⋅1 %, respectively). Research indicates that childhood weight status is a significant predictor of weight-related issues in later life^(3)^, though healthy eating interventions in childhood may serve as a protective factor against this outcome^(4)^. The UK Department of Health^(5)^ pledged to support efforts to reduce childhood obesity, with the target of a sustained downward trend in the incidence of childhood obesity by 2020. This target has not been met^(6)^, and there are at present renewed calls by the Government to tackle the nation's poor eating habits in light of the role that obesity plays in COVID-19 morbidity and mortality^(7)^. Indeed, global trends show that concerted action is required to combat poor nutrition and childhood obesity, and the British Medical Association (BMA)^(1)^ agrees that promoting healthier diets in children is a public health priority.

In the UK, the primary school environment is accessible to all children nationally and offers a convenient setting for implementing healthy eating interventions. Children spend most of their school day in classrooms where healthful behaviour can be encouraged via curricular activities, and lunch is served and consumed in the school cafeteria where key variables such as meal content and serving size can be controlled for those children who eat school-provided lunches^(8)^. Several school-based multicomponent interventions have been developed to target poor childhood nutrition in the UK schools, with some^(9,10)^ reporting moderate positive effects on children's consumption. Many ‘healthy school’ initiatives have been introduced regionally and nationally, although monitoring of their implementation and evaluation of their results on children's dietary habits and health outcomes remain patchy^(11)^.

The Food Dudes healthy eating programme for primary schools^(12,13)^ is an evidence-based multicomponent intervention that had been adopted regionally in the Midlands of England and nationally in the Republic of Ireland. It promotes fruit and vegetable consumption using the principle of the three ‘Rs’: role modelling, repeated tasting, and rewards. Role modelling has been acknowledged as a consistently effective tool for consumption behaviour modification^(14,15)^. Research has identified several factors that increase the likelihood of imitation, including observing a model's behaviour being rewarded^(16)^, the model being of a similar age or slightly older^(17)^, and multiple models being present^(18)^. The Food Dudes, presented in DVD episodes during the first stages of the intervention, are played by live actors and corresponding animated characters; they have been designed as role models that incorporate these elements. In addition to this, children are given many opportunities to taste fruit and vegetables over the course of the programme. Repeated tasting of a food item has been associated with increased expressed liking for, and consumption of, that food item^(19,20)^. The final factor is contingent delivery of rewards for tasting, then eating all the target foods. These prizes are carefully chosen to be affordable, desirable, and to indicate to the children that they are associated with a behaviour that is independently enjoyable and high-status, one that children should strive to repeat for its intrinsic benefits^(21)^. These three factors comprise the initial 4-week intensive phase of the intervention. More recently, the Food Dudes intervention has evolved to include dining room support, including behavioural nudges encouraging children to select healthy options, and online support and activities, as a ‘maintenance’ phase.

The Food Dudes programme has been shown to be successful in increasing fruit and vegetable consumption at snack time, lunchtime, and at home, particularly for those children who initially ate little-to-none of these food items at baseline^(12,13)^. The programme has also been found to be effective in a variety of primary school contexts, including in Ireland, where children take lunch with them into school requiring lunchbox food provision to be addressed^(12)^, cross-culturally in Italy^(22)^ and the US^(23)^, with pre-school children^(24,25)^, and in special schools for children with moderate to severe learning difficulties^(26)^. The programme had been adjusted in response to feedback from schools and commissioners and the updated version includes new multimedia materials, maintenance programme that changes choice architecture of school dining environment, and gamification of lunchtime fruit and vegetable consumption. The present study had been designed to extend previous findings with the evaluation of this updated intervention, using validated, sensitive methods of recording individual children's lunchtime eating.

In the existing literature, the researchers have typically used food diaries and frequency questionnaires^(9,10)^ to assess consumption to approximate servings, or the more reliable method of direct observation^(12,13)^ to measure differences in fruit and vegetable consumption to the nearest half- or quarter-portion. Though high levels of inter-rater agreement can be achieved^(12,13,27)^, and measures were more sensitive than standard dietary recall estimates in recording small but significant improvements^(11)^, no previous assessment of a multicomponent intervention has been able to consider changes to children's consumption at a more detailed level of macro- and micronutrients, or indeed investigate dietary change to the level of individual children. Instead, results were reported by cohort at the ‘portion’ or ‘serving’ level, which are not sufficiently sensitive to assess children's actual nutrient intake and the impact of any displacing influence that participation in multicomponent dietary programs may have on less healthful food consumption. In the present study, we have addressed these shortcomings.

## Method

### Aims

The present study examined changes in children's lunchtime consumption of fruit and vegetables, followed by the exploration of the effects of the Food Dudes programme on calorie consumption, and to a macronutrient (fat, saturated fat, protein, carbohydrates, sugar, and fibre) and micronutrient (sodium, potassium, vitamin C, and vitamin E) level. Participants’ consumption of selected micronutrients was investigated for multiple reasons: sodium and potassium to monitor any differences in salt consumption; vitamin C because of its association with fruit and vegetables, and vitamin E because of research indicating that European children may be deficient in it^(28)^.

### Participants

Children from two primary schools in Central Leeds, randomly allocated to the experimental conditions, received information regarding their school's intention to take part in the present study, including an opt-out consent form. No pupils chose to opt-out of study participation, leaving an intervention school sample of 78 children (year 1 *n* 6; year 3 *n* 30; year 5 *n* 22), and a control school sample of 75 children (year 1 *n* 25; year 3 *n* 29; year 5 *n* 21). Both samples were balanced on sex, and matched on size, deprivation status, Ofstead report^(29)^, percentage ethnic minority, and school dinner provision (Leeds Catering).

### Materials

To collect consumption data, we used four digital cameras and standardised their placement using tripod stands, tape measures, and protractors. Food items were displayed on paper plates (lunchbox meals), plastic school trays, or school plates. Self-adhesive identification labels were attached to each participant's school jumper and to their lunchbox or lunch tray/plate for later coding from photographs.

### Procedure

#### Data collection

Data were recorded over four consecutive days (Monday–Thursday) at two time points (baseline and follow-up) spaced 2 months apart. The protocol was identical at both time points. School lunch menus were matched across the two measurement occasions with the exception that, in the intervention school follow-up, vegetable-rich main courses and fruit-based desserts replaced the previously offered foods twice a week.

The digital photography method validated in previous research^(27)^ was used. In the morning, researchers collected lunchboxes: removed, photographed, and replaced their contents. Lunchbox items were not weighed, as weight could be estimated from manufacturer information, or average weights of similar products (e.g. satsumas and ‘fun’-sized apples are approximately equal sized due to cosmetics standards of fruit and vegetables sold in supermarkets). For school lunch meals, average portion weights were calculated from five servings of each food item. At lunchtime, researchers collected pre-consumption photographs of trays/plates, before allowing children to sit and eat their lunch as usual. Once participants had finished eating, they handed their lunchbox or tray/plate to researchers, who took post-consumption photos of each participant's lunch waste.

#### Intervention procedure

Phase 1 of the intervention was teacher-led and conducted in children's classrooms. Teachers were trained by intervention specialists, who visited the school intermittently throughout intervention implementation, to ensure intervention fidelity. Every day, children either watched an episode of the Food Dudes, which combined live action and animation to show the Dudes battle the Junk Punks, villains with appalling dietary habits; or were read a letter from the Dudes. These cool characters modelled fruit and vegetable consumption and urged the children through words and songs to be like them. At the same time, children were presented with one portion of fruit and one portion of raw vegetable each in their classrooms. During the first 4 d of Phase 1, they received a reward for simply tasting each of the presented food items; thereafter, they had to eat the full portions to receive a prize (small branded items of stationery and toys). Over 16 d, four pairings of fruit and vegetables were presented at mid-morning snacktime four times each, on a rolling schedule. Children also recorded the fruit and vegetables that they ate at home and received prizes for completing these home diaries.

During Phase 2, which lasted until the end of the school year (4 months), the intervention moved to the dining room. Children were encouraged to bring more fruit and vegetables from home or to choose them from the school menus. They were given collectable ‘level cards’ in lieu of immediate tangible rewards, which operate in a similar way to loyalty stamp cards. To complete a card, children were required to eat a specified number of portions of fruit and vegetables (which increased across levels) and have this behaviour verified by a Food Dudes hall monitor, a child in an older year group given the responsibility of monitoring fruit and vegetable consumption via direct observation. Once a ‘level card’ had been completed, it could be exchanged for a tangible reward.

This phase also included changes to the choice architecture of the dining room to facilitate fruit and vegetable selection for children buying school lunches: (1) increasing the variety provided by the school on ‘Special Energy Days’ (Tuesdays and Thursdays) by substituting foods high in fat, salt, and sugar with healthier meal options on the lunchtime menu (for example, recipes were provided by the Food Dudes to increase the vegetable content of composite meals, and puddings were replaced with fruit and yoghurt); (2) changing the order of serving in the canteen so that fruit and vegetables were offered before other starchy options and presenting them in an attractive way; (3) cueing consumption via branding; (4) encouraging adults and older children to be healthy eating role models, and (5) providing catering staff with training in encouraging the consumption of fruit and vegetables through subtle nudges.

No changes were made to the children's classroom or dining room routines in the control school.

#### Data processing and coding

##### Determining the final sample

To be included in the analysis, participants were required to have data for at least 2 d per time point, including at least one ‘normal’ day and one ‘special energy’ day in the intervention school. Although there were no special days in the control school, this procedure was applied equally, leaving 116 participants (58 from each school), 77 % of the consented sample. In the intervention school, 40 % of the sample were female and 45 % were key stage 1 (KS1; younger) children; in the control school, 66 % were female and 48 % were from KS1.

##### Calculating children's consumption of each food in grams

The consumption of each food item was estimated from servings and plate waste to the nearest 10 % increment on an 11-point scale (0–100 %) by the lead researcher and one research assistant, before being converted to a gram weight. Inter-rater reliability, calculated on 20 % of the data, was high (Cohen's *k* 0⋅934; CI 0⋅912, 0⋅956). For school lunches, mean weights for each food item had been collected before lunchtime. For lunchboxes brought from home, weight for each food item was estimated by referring to product information published by the manufacturer or standardised tables.

##### Calculating overall consumption scores

Researchers calculated the nutritional content of the foods consumed using published product information, catering company recipes and McCance and Widdowson's The Composition of Foods Seventh Summary Edition^(30)^, a comprehensive manual detailing the macro- and micronutrient content of the most regularly consumed food items in the UK. A ‘global’ score, a total for the entire meal, was calculated for each variable (grams of fruit and vegetable consumed, calories and macronutrients (fat, saturated fat, protein, carbohydrates, sugar, and fibre) and micronutrients (sodium, potassium, vitamin C, and vitamin E) on each day, then averaged across baseline and follow-up for each participant.

##### Preliminary analyses

Distribution tests showed that much of the data were either skewed or kurtosed or both; therefore, non-parametric analyses were conducted throughout. No outliers have been removed; variability is an expected feature of children's consumption data. There were no sex differences in either the baseline consumption or magnitude of the observed changes. Older children consumed more calories than their younger peers, but this difference was small and our two samples were well matched in age.

##### Other considerations

The Food Dudes programme had been designed to increase fruit and vegetable consumption in children, and this was the primary outcome measured in the present study; therefore, trial details did not need to be registered in advance. Macro- and micronutrient analyses that are most commonly included in similar nutritional evaluations were performed and are reported in full. Given the multiplicity of tests, effect sizes as well as *P*-values are reported throughout.

## Results

Mixed-effects repeated measures maximum-likelihood regression models with fixed effects (Time) and AR(1) heterogeneous covariance matrix were run to control for any possible clustering effects on the scores for the dependent variables for the intervention and control condition in SPSS-24^(31)^. Model estimates for the mixed effect analyses (baseline and follow-up within-group comparisons) are presented in Supplementary Appendix 1 of Supplementary material.

All between-groups comparisons were analysed using Mann–Whitney *U* tests. Effect sizes were calculated for Mann–Whitney *U* tests by dividing the *z*-score by the square root of the number of observations, with the subsequent *r* value indicating the magnitude of the effect (0⋅1–0⋅29 = small, 0⋅3–0⋅49 = moderate and ³0⋅5 = large effect)^(32)^. For each figure presenting box plots of the results, medians, interquartile ranges and distributions of children's consumption are shown at baseline and follow-up for the intervention and control schools.

### Changes in children's fruit, vegetable, protein and calorie consumption

#### Fruit

**Fig. 1.**
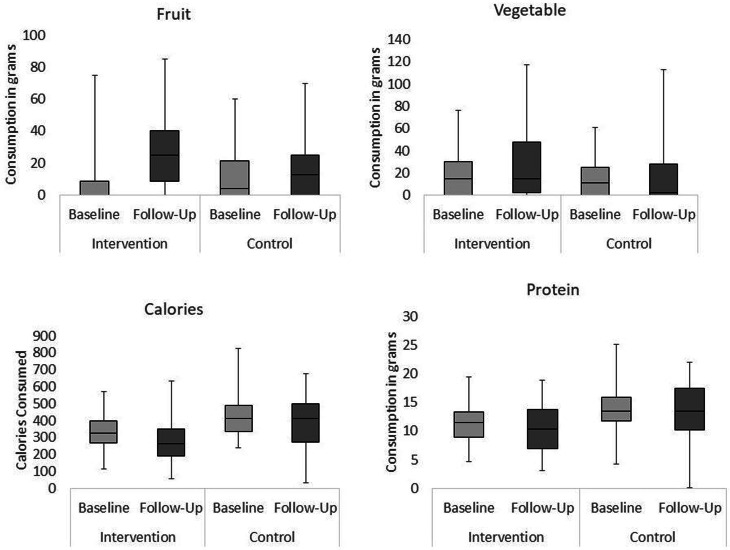
Changes in children's fruit, vegetable, calorie and protein consumption across time (baseline and follow-up) in each experimental condition (intervention and control).

Fig. 1 shows that a significant increase in fruit consumption with a large effect size was observed in the intervention school (*Mdn difference* 20 g, *F*(1, 57) 30⋅18, *P* < 0⋅001), while consumption remained stable in the control school (*Mdn difference* 0 g, *F*(1, 57) 3⋅15, *P* = 0⋅081). At baseline, the two experimental conditions were not perfectly matched; children in the control school consumed more fruit than the intervention school (*Mdn intervention* 0 g, *Mdn control* 4 g, *U* 1178⋅0, *P* = 0⋅003, *r* −0⋅40), but the children in the intervention school consumed more fruit than the control school at follow-up (*Mdn intervention* 25 g, *Mdn control* 14 g, *U* 1204⋅0, *P* = 0⋅008, *r* −0⋅36).

#### Vegetables

An increase with a moderate effect size was identified in the intervention school (*Mdn difference* 4 g, *F*(1, 57) 8⋅99, *P* < 0⋅01) but not in the control school (*Mdn difference* 0 g, *F*(1, 57) 0⋅37, *P* = 0⋅548). Vegetable consumption was matched across the two conditions at baseline (*Mdn intervention* 15 g, *Mdn control* 11 g, *U* 1535⋅0, *P* = 0⋅406, *r* −0⋅08), but at follow-up children in the intervention school consumed a significantly higher weight of vegetables (*Mdn intervention* 15 g, *Mdn control* 2⋅5 g, *U* 1251⋅0, *P* = 0⋅015, *r* 0⋅23).

#### Protein

A small decrease was observed in the intervention school (*Mdn difference* −2 g, *F*(1, 57) 5⋅22, *P* < 0⋅05) and in the control school (*Mdn difference* 0 g, *F*(1, 57) 2⋅06, *P* = 0⋅044). Children in the intervention school consumed less protein than those in the control school at both time points (baseline *Mdn intervention* 12 g, *Mdn control* 14 g, *U* 900⋅0, *P* < 0⋅001, *r* −0⋅41; follow-up *Mdn intervention* 10 g, *Mdn control* 14 g, *U* 1171⋅0, *P* = 0⋅005, *r* −0⋅27). At follow-up, children in both schools consumed over one-third of their guideline protein daily intake of 28 g, in line with dietary recommendations^(33)^.

#### Calories

Children in Key Stage 2 (KS2) generally consumed more calories than children in KS1 in both schools, with the exception of the control school at follow-up, where children in KS1 consumed significantly more calories than KS2 (see Table 1).

**Table 1.** Calories consumed by children in the two age subgroups, in the two conditions, at the two time points

Condition	Baseline	Follow-up
Intervention	KS1 *Mdn* 307⋅11 sd 93⋅33	KS1 *Mdn* 210⋅45 sd 132⋅23
	KS2 *Mdn* 374⋅03 sd 90⋅24	KS2 *Mdn* 325⋅26 sd 124⋅68
	*U* 241⋅0, *P* = 0⋅006	*U* 282⋅0, *P* = 0⋅036
Control	KS1 *Mdn* 348⋅61 sd 88⋅82	KS1 *Mdn* 434⋅80 sd 113⋅95
	KS2 *Mdn* 432⋅48 sd 93⋅33	KS2 *Mdn* 365⋅98 sd 191⋅47
	*U* 275, *P* = 0⋅024	*U* 242⋅0, *P* = 0⋅006

There was a significant decrease between baseline and follow-up in the intervention school (*Mdn difference* −46⋅51, *F*(1, 57) 16⋅39, *P* < 0⋅001) but not in the control school (*Mdn difference* 3⋅95, *F*(1, 57) 2⋅77, *P* = 0⋅102). Schools were not perfectly matched, with the control children consuming significantly more calories than the intervention group at baseline (*Mdn intervention* 323⋅16, *Mdn control* 412⋅21, *U* 959⋅0, *P* < 0⋅001, *r* −0⋅37) and at follow-up (*Mdn intervention* 260⋅91, *Mdn control* 413⋅82, *U* 1045⋅0, *P* < 0⋅001, *r* −0⋅33).

### Other changes in children's macronutrient consumption

#### Carbohydrates

**Fig. 2.**
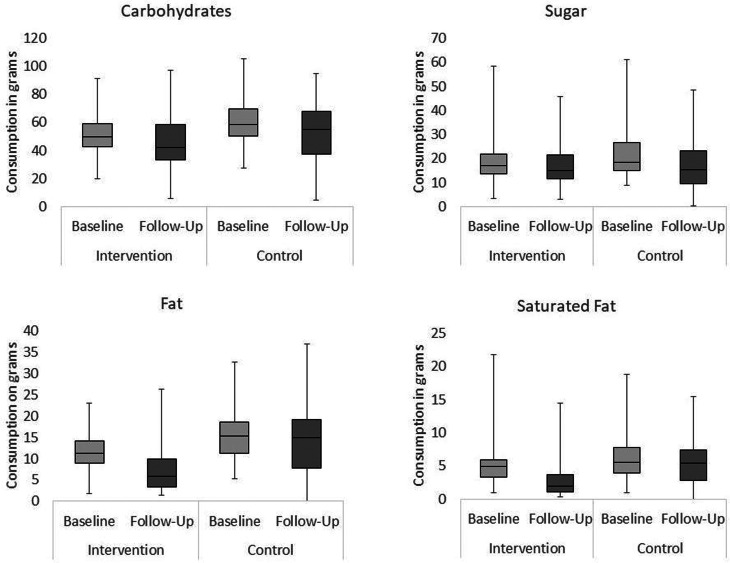
Changes in children's intake of carbohydrates, sugar, fat and saturated fat over time (baseline and follow-up) in each experimental condition (intervention and control).

Fig. 2 shows that carbohydrate consumption remained stable over time in the intervention school (*Mdn difference* −4 g, *F*(1, 57) 3⋅93, *P* = 0⋅052), but a significant decrease was observed in the control school (*Mdn difference* −4 g, *F*(1, 57) 6⋅50, *P* = 0⋅014). Carbohydrate consumption was higher in the control school at baseline (*Mdn intervention* 50 g, *Mdn control* 58 g, *U* 1043⋅0, *P* < 0⋅001, *r* −0⋅33) but matched at follow-up (*Mdn intervention* 42 g, *Mdn control* 55 g, *U* 1366⋅0, *P* = 0⋅081, *r* −0⋅16), probably due to large variability and consequent overlap of distributions.

#### Sugar

Participants’ sugar intake was assessed in total, without segregating sugars from fruit and vegetables. This was in order to address any concerns regarding increased sugar consumption due to increased fruit consumption. There was no change over time in the intervention school (*Mdn difference* −1 g, *F*(1, 57) 0⋅56, *P* = 0⋅456), but a decrease was recorded in the control school (*Mdn difference* −4 g, *F*(1, 57) 8⋅33, *P* = 0⋅006). The latter represented 10 % of recommended daily maximum intake^(33)^. As before, these results may be associated with large variability in the data set. Between conditions, sugar consumption was matched at baseline (*Mdn intervention* 17 g, *Mdn control* 18 g, *U* 1408⋅0, *P* = 0⋅131, *r* −0⋅14) and follow-up (*Mdn intervention* 15 g, *Mdn control* 15 g, *U* 1640⋅0, *P* = 0⋅817, *r* −0⋅02).

#### Fat

While consumption in the intervention school decreased significantly over time (*Mdn difference* −5 g, *F*(1, 57) 30⋅66, *P* < 0⋅001), consumption in the control school remained stable (*Mdn difference* 0 g, *F*(1, 57) 1⋅83, *P* = 0⋅182). Fat consumption was higher in the control school at baseline (*Mdn intervention* 11 g, *Mdn control* 15 g, *U* 1010⋅0, *P* < 0⋅001, *r* −0⋅35) and at follow-up (*Mdn intervention* 6 g, *Mdn control* 15 g, *U* 854⋅5, *P* < 0⋅001, *r* −0⋅43).

#### Saturated fat

A decrease was observed in the intervention school (*Mdn difference* −2 g, *F*(1, 57) 6⋅74, *P* < 0⋅05), but no change was seen in the control school (*Mdn difference* 2 g, *F*(1, 57) 1⋅62, *P* = 0⋅208). Consumption was matched at baseline (*Mdn intervention* 5 g, *Mdn control* 5 g, *U* 1398⋅0, *P* = 0⋅117, *r* −0⋅15), but at follow-up, participants in the intervention school consumed significantly fewer grams of saturated fat than participants in the control school (*Mdn intervention* 2 g, *Mdn control* 5 g, *U* 959⋅0, *P* < 0⋅001, *r* −0⋅38).

#### Fibre

No significant differences were observed over time or across conditions (Intervention Baseline *Mdn* 2⋅44 sd 1⋅03, Follow-Up *Mdn* 2⋅69 sd 1⋅41; Control Baseline *Mdn* 2⋅68 sd 0⋅81, Follow-Up *Mdn* 2⋅72 sd 2⋅82).

### Changes in children's micronutrient consumption

#### Sodium

**Fig. 3.**
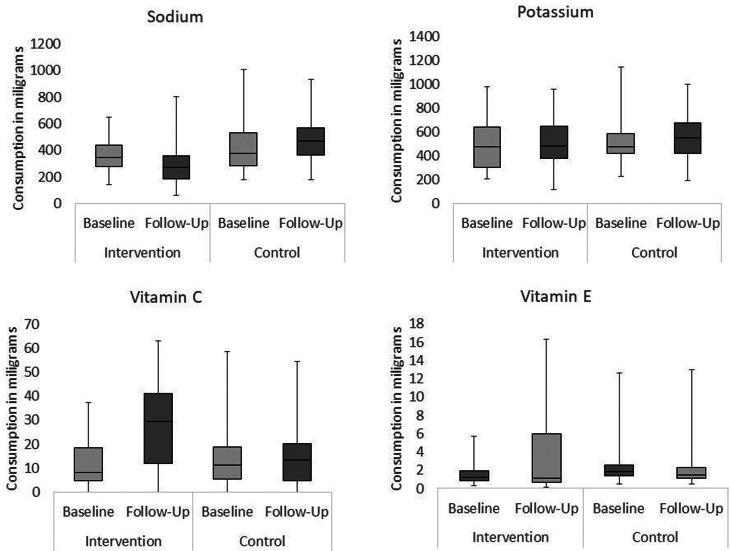
Changes in children's intake of sodium, potassium, vitamin C and vitamin E over time (baseline and follow-up) in each experimental condition (intervention and control).

Fig. 3 shows that a significant decrease was observed in the intervention school (*Mdn difference* −73 mg, *F*(1, 57) 10⋅20, *P* < 0⋅01). By contrast, a significant increase was observed in the control school (*Mdn difference* 93 mg, *F*(1, 57) 3⋅50, *P* = 0⋅067). Sodium consumption was matched between groups at baseline (*Mdn intervention* 343 mg, *Mdn control* 375 mg, *U* 1373⋅0, *P* = 0⋅088, *r* −0⋅16) but significantly higher in the control school at follow-up (*Mdn intervention* 270 mg, *Mdn control* 469 mg, *U* 597⋅0, *P* < 0⋅001, *r* −0⋅57). This 200 mg difference in average consumption represented 100 % of children's daily recommended sodium intake^(34)^.

#### Potassium

No significant differences were observed over time or across conditions (Intervention Baseline *Mdn* 476⋅66 sd 190⋅14, Follow-Up *Mdn* 480 sd 200⋅31; Control Baseline *Mdn* 472⋅84 sd 169⋅38, Follow-Up *Mdn* 551⋅48 sd 191⋅16).

#### Vitamin C

A significant 22 mg increase was observed over time in the intervention school (*Mdn difference* 19 mg, *F*(1, 57) 12⋅91, *P* < 0⋅01), representing over two-thirds of guideline minimum recommended intake^(35)^, while no difference was observed in the control school (*Mdn difference* 1⋅7 mg, *F*(1, 57) 1⋅03, *P* = 0⋅315). Consumption was matched at baseline (*Mdn intervention* 8 mg, *Mdn control* 11 mg, *U* 1553⋅0, *P* = 0⋅476, *r* −0⋅07), but at follow-up, the intervention group consumed more vitamin C (*Mdn intervention* 30 mg, *Mdn control* 13 mg, *U* 1030⋅0, *P* < 0⋅001, *r* −0⋅34).

#### Vitamin E

Children's consumption significantly increased in the intervention school (*Mdn difference* 0 mg, *F*(1, 57) 14⋅27, *P* < 0⋅001) but remained stable over time in the control condition (*Mdn difference* 0 mg, *F*(1, 57) 1⋅69, *P* = 0⋅202). It was not perfectly matched at baseline, as intervention school children consumed significantly less vitamin E than the control (*Mdn intervention* 1 mg, *Mdn control* 2 mg, *U* 992⋅0, *P* < 0⋅001, *r* −0⋅36), but no difference was detected follow-up (*Mdn intervention* 1 mg, *Mdn control* 1 mg, *U* 1480⋅5, *P* = 0⋅266, *r* −0⋅11), when participants in each group consumed, on average, approximately one-third of the recommended daily amount^(35)^.

### Secondary analyses: main effects by lunch type

Last, we investigated whether the consumption patterns differed between children who ate school lunches and those who brought their food from home. In the intervention school, 73 % of children brought their lunches from home and the remaining 27 % had school lunches on most or all of their assessment days. In the control school, 41 % of children brought food from home and 59 % ate school lunches.

Small samples and unequal group sizes necessitate cautious interpretation of the findings. Furthermore, we did not employ a mixed-effects cluster analysis so as to avoid partialing out the effects identified in the main analyses, or increasing the possibility of Type 1 and Type 2 errors through running multiple comparisons of the same data. Comparisons were made between groups at baseline and in the magnitude of the changes over time (if any). We looked at fruit, vegetable, calories, sodium and vitamin C consumption – the key effects identified in the main analyses. The change over time was assessed by calculating the difference between baseline and follow-up observation points for each variable. These data are summarised in Table 2.

**Table 2.** Significant within-groups differences by lunch type (home packed *v*. school lunches) in the intervention (I) and control (C) schools

	Baseline differences	Magnitude of change differences
Fruit	–	I (*U* 128, *P* = 0⋅030, *r* −⋅20)
Vegetable	I (*U* 131⋅5, *P* = 0⋅033, *r* −0⋅2)	–
	C (*U* 313, *P* = 0⋅012, *r* −0⋅23)	
Calorie	I (*U* 71, *P* = 0⋅001, *r* −0⋅32)	–
	C (*U* 193, *P* = 0⋅017, *r* −0⋅22)	
Sodium	I (*U* 106, *P* = 0⋅006, *r* −0⋅26)	I (*U* 94⋅5, *P* = 0⋅003, *r* −0⋅28)
	C (*U* 79, *P* < 0⋅001, *r* −0⋅42)	
Vitamin C	I (*U* 90, *P* = 0⋅002, *r* −0⋅29)	–

*Note:* Magnitude of change was calculated by subtracting consumption at baseline from consumption at follow-up.

#### Fruit

Children in both schools, for each lunch type, were matched on their fruit intake at baseline. In the intervention school, the participants who consumed school meals increased their consumption of fruit significantly more than those who brought a lunch box to school (school meal *Mdn increase* 24⋅25; lunch box *Mdn increase* 8⋅85). This may be because school lunch fruit provision was changed more readily than parental provision. No such effect was found over time in the control school.

#### Vegetables

A difference in consumption between different lunch types was observed at baseline for both schools (intervention school meal *Mdn* 19 g, lunchbox *Mdn* 0 g; control school meal *Mdn* 20 g, lunchbox *Mdn* 0 g). In the intervention school, participants who were provided with school meals consumed significantly more vegetables than those bringing lunch boxes from home. This may be because vegetables were provided with each school meal but not in all lunch boxes. In the control school, this trend was reversed, with participants who brought lunch boxes to school consuming more vegetables than those provided with school meals. This was unexpected; we noted that many lunchboxes in this school contained cucumber.

#### Calories

A significant difference between lunch types was found in both the intervention and control schools at baseline (intervention school meals *Mdn* 318⋅25, lunchbox *Mdn* 421⋅64; control school meals *Mdn* 392⋅70, lunchbox *Mdn* 455⋅59). This was expected, with research consistently indicating that lunchbox food items are more likely to be high in fat, sugar and salt compared with their school meal counterparts^(36)^.

#### Sodium

Significant differences were observed between lunch types, with those participants who consumed food brought from home consuming significantly more sodium than those participants who ate school dinners, in both conditions (intervention school meal *Mdn* 323 mg, lunchbox *Mdn* 471 mg; control school meal *Mdn* 325 mg, lunchbox *Mdn* 563 mg). In the intervention school, those children who ate school meals reduced their sodium intake more than those who brought lunchboxes from home (school meals *Mdn difference* 22 mg; lunchbox *Mdn difference* 1 mg).

#### Vitamin C

In the intervention school, participants who ate school dinners consumed significantly more vitamin C than did those participants who brought their lunch from home (intervention school meals *Mdn* 11 mg, lunchbox *Mdn* 1 mg).

## Discussion

The present study was the first to conduct a full nutritional analysis of the effects of a multicomponent UK-based healthy eating intervention, the Food Dudes programme, on children's lunches. The results show that the programme successfully increased fruit and vegetable consumption, coupled with a decrease in overall energy (calorie) intake, in the intervention school. In-depth macro- and micronutrient analysis identified that these changes were associated with decreases in fat, saturated fat, and sodium, as well as increases in vitamins C and E. These positive changes were not identified in the control school, suggesting that they can be attributed to the Food Dudes programme rather than seasonal differences in consumption between the two measurement points. These results have significant implications for national-level efforts to reduce the incidence of childhood obesity, and improve childhood nutrition, as recommended by the Department of Health^(5)^ and BMA^(1)^, and the methodology^(27)^ (and subsequent rich data yielded in the present study) may inform future researchers investigating the health impact of dietary changes in a cafeteria environment.

The effects of the programme may have substantial health implications for the children who take part. Increased fruit and vegetable consumption is consistently associated with health benefits, such as having a protective effect against cancers, coronary heart disease, and stroke^(37)^. It has also been associated with children consuming fewer high-energy snacks^(38)^, leading to a significant reduction in calorie intake. A decrease in calorie intake is associated with a decrease in incidence of, and a protective effect against, overweight and obesity^(39,40)^. Indeed, the median calorie reduction recorded over time in the intervention group was 47 calories. Though predictions of weight change are subject to individual and environmental differences and cannot be confidently asserted^(41)^, it has been suggested that a daily positive energy balance of 120 calories can result in a 50-kg weight increase over 10 years^(42)^. The present effect, even if it was confined to lunchtime and did not transfer to home, could protect the children from substantial weight gain. This evaluation ought to be replicated with measurements of children's consumption over the entire day, to confirm that children did not compensate by consuming more calories during other meals and snack times, and to ascertain whether further benefits of increased fruit and vegetable consumption were obtained at their home – even if this has to be measured in a less direct and precise manner.

The present nutritional analysis also highlighted other potential health implications. The macronutrient decreases in fat and saturated fat consumption may themselves serve as protective factors against childhood obesity; high-fat intake is associated with an increased likelihood of obesity in children^(43)^. When considering micronutrient changes, a 200 mg between-groups difference in average sodium consumption was observed at follow-up between the two cohorts, totalling almost 100 % of their daily recommended sodium intake^(44)^. This represents a significant health implication for the intervention group, as identified by Sacks *et al.*^(45)^ who reported that a decrease in dietary intake of sodium of this magnitude can result in a significant reduction in risk of hypertension. In addition, a 17 mg difference in vitamin C consumption was observed between groups at follow-up, a difference of almost 50 % of the daily recommended intake for vitamin C^(35)^, while a significant increase in Vitamin E was identified in the intervention group between baseline and follow-up, presenting potential implications for immune function^(35,46)^.

During the nutrient analysis, we also identified some unexpected effects. A significant decrease in protein was observed in both conditions, and the intervention group consumed significantly less protein than the control group during both observation points. This variation, with a median difference at follow-up of 3⋅3 g, was possibly due to small differences in school provisions and representing less than 10 % of guideline daily recommended protein intake^(47)^. Considering this, we do not believe that this result is associated with any health implications. Due to increases in fruit and vegetable intake, we had anticipated an increase in fibre consumption, but none was found, possibly because other foods in children's lunches also contained fibre, and variability was large. We had expected a decrease in sugar consumption; however, no significant differences were identified in the intervention group, but a significant decrease in sugar was observed in the control group, resulting in sugar consumption being matched at follow-up. This may be because, although unhealthy snack items were displaced, the sugar from these foods was substituted with intrinsic sugars found in fruit. This would still present a favourable outcome. The World Health Organisation^(48)^ suggests that, since these sugars have not been associated with poor health effects, they should not be considered when assessing dietary sugar. Unfortunately, it was not possible to analyse the two types of sugar separately because of the lack of sufficiently detailed information.

A further limitation of the present research is that it is possible that children selected their food, and ate differently, as a result of their behaviour being observed. Researchers did not test for an observer effect; however, potential observer effects were mitigated by not specifying to participants what we were measuring. Instead, if children asked, researchers responded that they ‘are interested in learning more about what children like to eat in school’. Furthermore, the present research may have been vulnerable to a reaction effect. Due to the short-term nature of data collection, we did not identify a reaction effect; however, future investigations should include a longer data-collection period in order to test any influence of initial reaction to the intervention, or to being observed, on consumption.

## Conclusions

Overall, the present results show that short-term changes in children's dietary habits, achieved over 2 months, can be robust and significant, and that increasing fruit and vegetable consumption by the Food Dudes programme leads to displacement of fat, sugar, and salt, for both those children who were eating school lunches, and others (a majority) who brought their food from home. In the latter case, changes in provision that enabled children to enjoy a healthier diet were also indicative of parental support and habit change. Future research ought to provide a long-term tracking of these effects because potential health benefits of dietary change can only be realised if this change is sustained over time.

The present study builds upon previous research in several ways. Existing research into the effectiveness of the multicomponent healthy eating interventions in primary schools have not used sufficiently sensitive methods to assess macro- and micronutrient changes over time^(9,10,12,13)^. Using a previously validated digital photography methodology^(27)^, we were able to estimate food item consumption to a finer-grained scale, to the gram. Further to this, we were also the first to consider the impact on global nutrient consumption at lunchtime, rather than portion size consumption differences alone. These two factors allowed us to conduct a better and more detailed nutritional analysis of all food items consumed at lunchtime, yielding information regarding calorie, macro- and micronutrient consumption. This enabled us to assess the potential health benefits of participation in the Food Dudes programme, and verify a displacing effect of fruit and vegetable consumption on less healthy food items. To our knowledge, this is the first time that a detailed evaluation of this kind had been applied to a school-based intervention.

## Supplementary material

For supplementary material accompanying this paper visit http://dx.doi.org/10.1017/jns.2021.31. click here to view supplementary material
