# Supplementary material for: Changes in the nutritional content of children's lunches after the Food Dudes healthy eating programme
Source: J Nutr Sci. 2021 May 31;10:e40. doi: 10.1017/jns.2021.31 (PMC8342193; doi:10.1017/jns.2021.31)
Supplement: Supplementary file 1 [file S2048679021000318sup001.docx]

## **Appendix 1.**

Model estimates for the mixed effects analysis of the intervention condition, consumption variables.

| Dependent Variable | Estimate of Fixed Effect | *Std.Error* | *t* | *p* | ARH1 rho (std.error) | Wald Z (*p*-value) |
| --- | --- | --- | --- | --- | --- | --- |
| Fruit | -21.78 | 3.98 | -5.49 | <.001 | .003 (.130) | 0.03 (.979) |
| Vegetables | -9.91 | 3.30 | -2.99 | <.01 | .567 (.090) | 6.36 (.001) |
| Protein | 1.03 | 0.45 | 2.29 | <.05 | .553 (.090) | 6.08 (.001) |
| Calories | 52.06 | 12.86 | 4.05 | <.001 | .656 (.070) | 8.78 (.001) |
| Carbohydrates | 3.88 | 1.96 | 1.98 | .052 | .659 (.074) | 8.88 (.001) |
| Sugar | 1.14 | 1.52 | 0.75 | .456 | .208 (.126) | 1.66 (.098) |
| Fat | 4.06 | 0.73 | 5.54 | <.001 | .520 (.096) | 5.43 (.001) |
| Saturated Fat | 2.08 | 0.80 | 2.60 | <.05 | .551 (.091) | 6.03 (.001) |
| Sodium | 69.83 | 21.87 | 3.19 | <.01 | .251 (.123) | 2.05 (.040) |
| Potassium | -25.96 | 26.62 | -0.98 | .333 | .452 (.104) | 4.33 (.001) |
| Vitamin C | -13.06 | 3.63 | -3.59 | <.01 | .026 (.131) | 0.20 (.842) |
| Vitamin E | -2.24 | 0.60 | -3.78 | <.001 | .105 (.129) | 0.81 (.418) |

Model estimates for the mixed effects analysis of the marginal means for the intervention condition, consumption variables.

| Dependent Variable | T1 mean | T2 mean | Mean difference  (std.error) | *p* | 95% CI |
| --- | --- | --- | --- | --- | --- |
| Fruit | 6.04 | 27.83 | -21.78 (3.97) | <.001 | -29.72 to -13.85 |
| Vegetables | 18.15 | 28.06 | -9.91 (3.31) | <.01 | -16.53 to -3.30 |
| Protein | 11.34 | 10.31 | 1.03 (0.45) | <.05 | 0.13 to 1.03 |
| Calories | 331.80 | 279.73 | 52.06 (12.86) | <.001 | 26.32 to 77.70 |
| Carbohydrates | 49.33 | 45.45 | 3.88 (1.96) | .052 | -.03 to 7.80 |
| Sugar | 18.63 | 17.47 | 1.14 (1.52) | .456 | -1.91 to 4.19 |
| Fat | 11.61 | 7.58 | 4.06 (0.73) | <.001 | 2.59 to 5.52 |
| Saturated Fat | 5.92 | 3.84 | 2.08 (0.80) | <.05 | 0.48 to 3.68 |
| Sodium | 355.22 | 285.38 | 69.83 (21.87) | <.01 | 26.06 to 113.61 |
| Potassium | 482.08 | 508.04 | -25.96 (26.62) | .33 | -79.25 to 27.32 |
| Vitamin C | 15.55 | 28.61 | -13.06 (3.64) | <.01 | -20.34 to -5.79 |
| Vitamin E | 1.49 | 3.73 | -2.24 (0.59) | <.001 | -3.43 to -1.06 |

*Note*: 95% CI = 95% confidence intervals for lower and upper bound from the Bonferonni pairwise comparison for baseline to follow-up.

Model estimates for the mixed effects analysis of the control condition, consumption variables.

| Dependent Variable | Estimate of Fixed Effect | *Std.Error* | *t* | *p* | ARH1 rho (std.error) | Wald Z (*p*-value) |
| --- | --- | --- | --- | --- | --- | --- |
| Fruit | -7.26 | 4.09 | -1.77 | .081 | 0.06 (0.13) | 0.44 (.658) |
| Vegetables | -1.54 | 2.55 | -0.60 | .548 | 0.65 (0.08) | 8.58 (.001) |
| Protein | 3.04 | 1.47 | 2.06 | <.05 | -0.13 (0.13) | -1.03 (.304) |
| Calories | 50.57 | 30.41 | 1.66 | .102 | -0.21 (0.13) | -1.69 (.091) |
| Carbohydrates | 9.58 | 3.76 | 2.55 | <.05 | 0.06 (0.13) | 0.42 (.672) |
| Sugar | 5.56 | 1.93 | 2.89 | <.01 | 0.22 (0.13) | 1.73 (.083) |
| Fat | 1.85 | 1.37 | 1.35 | .182 | -0.08 (0.13) | -0.60 (.548) |
| Saturated Fat | 0.81 | 0.51 | 1.27 | .208 | 0.19 (0.13) | 1.50 (.134) |
| Sodium | -55.71 | 29.78 | -1.87 | .067 | 0.16 (0.13) | 1.25 (.211) |
| Potassium | -54.37 | 27.52 | -1.98 | .053 | 0.33 (0.19) | 2.79 (.005) |
| Vitamin C | -3.09 | 3.05 | -1.01 | .315 | 0.28 (0.12) | 2.25 (.024) |
| Vitamin E | -1.20 | 0.93 | -1.29 | .202 | 0.20 (0.13) | 1.60 (.110) |

Model estimates for the mixed effects analysis of the marginal means for the control condition, consumption variables.

| Dependent Variable | T1 mean | T2 mean | Mean difference  (std.error) | *p* | 95% CI |
| --- | --- | --- | --- | --- | --- |
| Fruit | 12.01 | 19.27 | -7.26 (4.09) | .081 | -15.46 to 0.93 |
| Vegetables | 16.09 | 17.63 | -1.54 (2.55) | .548 | -6.65 to 3.57 |
| Protein | 15.52 | 12.48 | 3.04 (1.47) | <.05 | 0.09 to 5.98 |
| Calories | 424.15 | 373.58 | 50.57 (30.41) | .102 | -10.32 to 111.46 |
| Carbohydrates | 60.59 | 51.01 | 9.58 (3.76) | <.05 | 2.06 to 17.10 |
| Sugar | 22.65 | 17.09 | 5.56 (1.93) | <.01 | 1.70 to 9.43 |
| Fat | 15.77 | 13.92 | 1.85 (1.37) | .182 | -0.89 to 4.59 |
| Saturated Fat | 6.49 | 5.67 | 0.81(0.64) | .208 | -0.47 to 2.09 |
| Sodium | 420.95 | 476.66 | -55.71 (29.78) | .067 | -115.35 to 3.92 |
| Potassium | 507.70 | 562.07 | -54.37 (27.52) | .053 | -109.48 to 0.74 |
| Vitamin C | 14.81 | 17.90 | -3.09 (3.05) | .315 | -9.20 to 3.02 |
| Vitamin E | 2.18 | 3.38 | -1.20 | .202 | -3.06 to 0.66 |

*Note*: 95% CI = 95% confidence intervals for lower and upper bound from the Bonferonni pairwise comparison for baseline to follow-up.
